# How the Environment Shapes Tactile Sensing: Understanding the Relationship Between Tactile Filters and Surrounding Environment

**DOI:** 10.3389/frobt.2022.930405

**Published:** 2022-07-11

**Authors:** Leone Costi, Perla Maiolino, Fumiya Iida

**Affiliations:** ^1^ Bio-Inspired Robotics Laboratory, Department of Engineering, University of Cambridge, Cambridge, United Kingdom; ^2^ Oxford Robotics Institute, University of Oxford, Oxford, United Kingdom

**Keywords:** tactile filters, tactile sensing, soft sensing, environment interaction, embodied intelligence, morphological computation, soft robotics

## Abstract

The mechanical properties of a sensor strongly affect its tactile sensing capabilities. By exploiting tactile filters, mechanical structures between the sensing unit and the environment, it is possible to tune the interaction dynamics with the surrounding environment. But how can we design a good tactile filter? Previously, the role of filters’ geometry and stiffness on the quality of the tactile data has been the subject of several studies, both implementing static filters and adaptable filters. State-of-the-art works on online adaptive stiffness highlight a crucial role of the filters’ mechanical behavior in the structure of the recorded tactile data. However, the relationship between the filter’s and the environment’s characteristics is still largely unknown. We want to show the effect of the environment’s mechanical properties on the structure of the acquired tactile data and the performance of a classification task while testing a wide range of static tactile filters. Moreover, we fabricated the filters using four materials commonly exploited in soft robotics, to merge the gap between tactile sensing and robotic applications. We collected data from the interaction with a standard set of twelve objects of different materials, shapes, and textures, and we analyzed the effect of the filter’s material on the structure of such data and the performance of nine common machine learning classifiers, both considering the overall test set and the three individual subsets made by all objects of the same material. We showed that depending on the material of the test objects, there is a drastic change in the performance of the four tested filters, and that the filter that matches the mechanical properties of the environment always outperforms the others.

## 1 Introduction

The mechanical characteristics of any given structure greatly affect and alter the capability to sense and process tactile information (Scimeca et al., 2018; Hughes and Iida, 2017) and, therefore, affect the performance of tactile-driven tasks (Costi et al., 2021a). Biological systems show us that the mechanical characteristics of a sensor should be appropriate to the task or environment in which the sensor operates. Nature has developed task-specific sensors and sensing networks for millions of years (Rawlings et al., 2012), developing systems composed of different sensing units, called receptors, embodied in a great variety of passive mechanical structures, called filters. A filter is any mechanical structure that is placed between the sensing unit and the environment and can have a varying morphology, spatial distribution, and density, perfectly tuned based on the main task they have to perform to obtain the best possible performance (Vallbo and Johansson, 1984; Maeno et al., 1998). The most common tactile filter observable in nature is the skin (Lewin and Moshourab, 2004), which clearly shows how the mechanical characteristics of such soft tissue change depending on the main task of a given body part (Barlow, 1987). However, the scale and complexity used by nature are still out of technology’s reach, preventing us from having skin-like performance when it comes to tactile data acquisition and classification (Iida and Nurzaman, 2016).

Previous studies in the field of filter-based tactile sensing have focused on the effect of the filter’s morphology on the structure and quality of the recorded data, investigating the information gain’s maximization (Thuruthel et al., 2020), the amplification of the sensor’s sensitivity (Fend et al., 2004), task-specific optimization (Qi and Hirai, 2019), and the relationship between redundancy and localization error (Costi et al., 2021b). Moreover, researchers have deeply studied the relationship between the morphology of the sensor and the action for perception (Huang et al., 2019; Scimeca et al., 2020b, 2021). From their results, it emerges how in action–based perception is strongly affected by the morphology of the tactile filter (Bernth et al., 2018) and the inevitable trade-off when selecting the material of the filter. Considering the filter’s hardness as an example, softer interfaces are more compliant (Brown et al., 2010; Margheri et al., 2012; Pfeifer et al., 2014; Hinitt et al., 2015) and can provide better adhesion to the object of interest (Trinh and Shibuya, 2019; Qi et al., 2020), therefore, increasing the quality of tactile information thanks to a larger contact area. On the other hand, a soft filter behaves as a ‘mechanical low-pass filter’, perturbating the data and filtering out high-frequency components (Shimojo, 1997). However, the role of the environment in dictating what the best filter’s properties are has often been overlooked. We believe that the environment plays a major role in determining the quality of the tactile data and has a strong influence on the optimal characteristics of the tactile filter.

In order to avoid the trade-off presented by static design for tactile filters, some researchers have developed dynamic filters, able to change properties online as they gather information from the environment. Noticeable examples include the usage of hot melt adhesive to reshape the sensor morphology (Nurzaman et al., 2013) and of liquid sensors able to tune dynamic range and sensitivity (Liao et al., 2015). Some studies focused precisely on online stiffness adaptation (Hughes et al., 2021; Costi et al., 2022b), highlighting how strongly the mechanical properties of the filter’s material can influence the embodied intelligence of the filter itself and, in turn, the task of tactile sensing. However, once again, little to no attention was given to the role of the environment. All in all, researchers have tried to come up with an optimal solution for tactile filtering, exploiting both static and dynamic designs, without considering what role the environment and the surrounding objects play in the task of tactile classification.

Soft robots and soft sensors are manufactured in a very wide range of materials and elastomers (Lee et al., 2017), making it challenging to generalize the possible advantages and disadvantages obtained by the material’s choice in relation to tactile sensing. However, a recent attempt at creating a more solid framework for the field (Marechal et al., 2021) highlighted that the vast majority of soft robots nowadays are manufactured using two main silicone rubber series: Ecoflex (*Smooth-On*), mainly used for extreme deformations and induced mechanical instabilities such as ballooning (Herzig et al., 2021; Costi et al., 2022a), and Dragon Skin (*Smooth-On*), used to create more solid structures and slower controlled deformations (Gao et al., 2021).

The aim of this work was to analyze the effect of the environment on tactile information-based object classification. We wanted to show how the ideal filter’s mechanical properties are related to the environment’s ones. Moreover, in order to generalize our findings and focus this study on soft robotics applications, we used, as the filter’s materials, the four most used elastomers in soft robotics, from most to least compliant: Ecoflex 00–10, Ecoflex 00–30, Dragon Skin 10, and Dragon Skin 30. To do so, we manufactured four filters of the same geometry and different material, and we used a sensorized UR5 robotic arm (*Universal Robots*) mounting the different filters to perform active touching on a set of 12 standardized objects of varying materials, shapes, and textures. We showed how the different materials affect the structure of the collected data and how such data perform when used in a tactile classification task. First, data were characterized in terms of variance retention and clustering behaviors. Then, they were used as input for nine standard machine learning classification algorithms. The same analysis was carried out separating the test set into three subsets based on the objects’ material. Finally, we proved that matching the environment’s mechanical properties is the best strategy to optimize data quality and tactile classification accuracy while operating with static filters.

In the remainder of the article, we describe the manufacturing of the filters and the implementation of the overall sensing system and testing bench in Section 2, followed by the results in Section 3 and the discussion and final remarks in Section 4.

## 2 Materials and Methods

### 2.1 System Overview and Manufacturing

**FIGURE 1:**
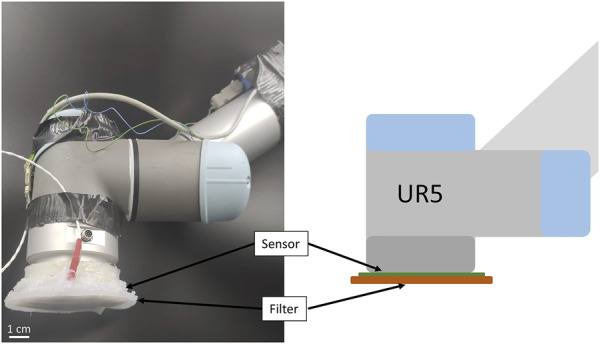
On the left, implementation of the active touching system composed of UR5 robotic arm, tactile sensor, and tactile filter. On the right, schematics of the system.

A UR5 robotic arm was used to perform active touch on a set of known standard objects (see Figure 1). The arm carried both the sensing unit and the tactile filter on the end-effector.

**FIGURE 2:**
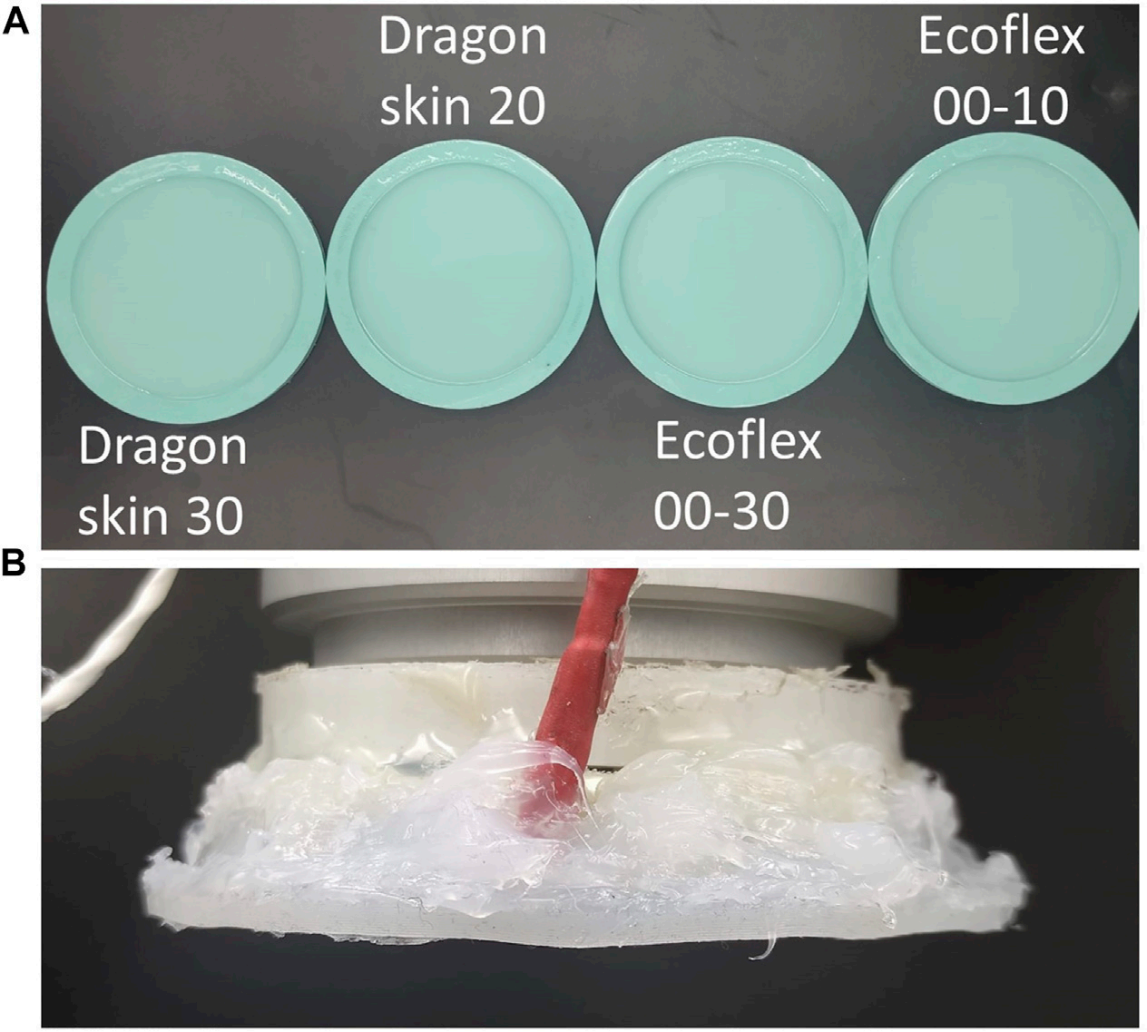
Fabricated elastomeric filters in different materials **(A)** and implementation of the filter on the end-effector of the robotic arm **(B)**.

The tactile sensor was a capacitive sensor disk composed of 50 “taxels”, providing high sensitivity and spatial distribution over the surface of the sensor. The sensor had a diameter of 80 *mm*, and the taxels were uniformly distributed on its surface. They had a resolution of 16 bits corresponding to a variation of capacitance, which was proportional to the pressure acting on top of it. Therefore, they were able to detect normal load only. Details of the specific sensor and its fabrication have been previously reported (Maiolino et al., 2013). The tactile filters were manufactured using a silicone casting technique in PLA (*Polymaker*) moulds, 3D printed with a Pro 2 printer (*Raise3D*) for each filter, and the 2 phases were manually mixed in a 1:1 ratio for 5 min and then placed in the vacuum pump for 20 min. Finally, the silicone was cast into the molds, and excess material was removed through tape casting using a doctor blade (see Figure 2A). The four selected materials span a wide range of shore hardness, with the two Dragon Skin materials showcasing 30 and 10*A* and the Ecoflex ones 00– 30 and 00– 10 in the ASTM D-2240 hardness scale, effectively covering the entire spectrum of mechanical properties of the elastomers used in soft robotics. In real-world applications, the material of the filter is normally within a range of materials dictated by the given task. In the attempt of producing results that can be generalized and applied widely in soft robotics, we selected such materials due to their presence in most soft robotics systems. The filters were attached to the sensing unit using Sil-poxy (*Smooth-On*): the silicone glue was applied only on the sides of the sensor to avoid any additional filtering that would have resulted from placing it on top of the taxels (see Figure 2B). In doing so, we ensured that the interface between sensor and filter was affected by just two factors: the mechanical properties of the filter and the ones of the sensor, which was the same for all four tested filters. During the experimental trials, the end-effector was placed above the selected object, and then the acquisition of tactile data was performed in two steps: the approaching phase and the touch phase. First, the filter was moved down until contact with the environment, and then a planar “rubbing motion” was performed (see Section 2.2). This process is also known as active touch because the system actively reaches and touches the environment, which is passive.

### 2.2 Experimental Protocol

In order to test the effect of the different materials on tactile classification tasks, we collected tactile data on a standardized set of 12 objects made of 3 different materials, showcasing different shapes and textures for each tested material.

Once the object to be touched was sampled, the tactile sensing process was performed in two phases: the approaching phase and the touch phase. In the approaching phase, the end-effector was lowered in order to achieve contact with the object of interest. During this phase, the end-effector was moved normally downward until the capacitive tactile sensor at its extremity detected a touch event. A touch event is defined as a rise by more than 5% of their reading range in any of the 50 taxels. Note that the aforementioned rise in pressure value has to be detected by the sensing unit; therefore, it is the signal after the effect of the tactile filter: different filter’s materials can behave mechanically differently and, in turn, achieve different levels of interaction with the environment. The touch phase consisted of a 5 s interaction in which the robot performed a ‘rubbing motion’ by moving 2 *mm* along *x* and 2 *mm* along *y* on the plane in which contact was detected. During this phase, the sensor was sampled at 50 *Hz*, retrieving a total of 250 tactile maps for each trial, composed of a value for each one of the 50 different taxels. As a result, each collected data point corresponding to a single trial, had 12,500 dimensions.

**FIGURE 3:**
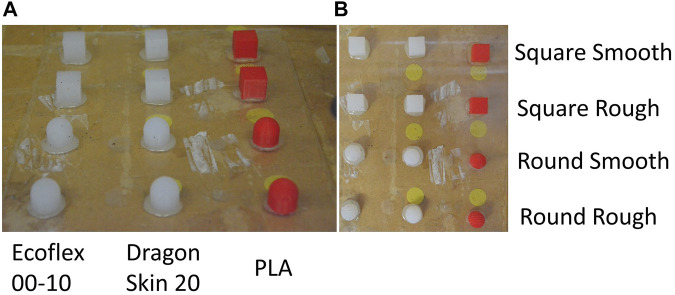
Side view **(A)** and top view **(B)** of different objects used for the data acquisition. From left to right: Ecoflex 00–10, Dragon Skin 20, and PLA. From top to bottom: Smooth Square, Rough Square, Smooth Round, and Rough Round.

Concerning the set of test objects, we decided to use 12 objects with different mechanical characteristics: three different materials, two different shapes, and two different textures (see Figure 3). The three materials were PLA, Dragon Skin 20, and Ecoflex 00–10: PLA objects were directly 3D printed, whereas elastomeric ones were the result of silicone casting in 3D printed PLA molds. The fabrication process was the same, as previously described in Section 2.1, both for PLA 3D printing and silicone casting. The two different shapes were designed as follows: the squares were 20 *mm* edge cubes, and the rounds were 20 *mm* diameter and 10 *mm* height cylinders with 20 *mm* diameter half-spheres on top. Finally, the rough objects were ridged by adding 1 *mm* deep and 1 *mm* wide grooves at a distance of 1 *mm*. The objects’ material choice allowed us to investigate the filters’ behavior when interacting with an environment of the same material, of a different material of the same series, and of a different silicone series, as well as when interacting with a generic rigid environment (PLA). Overall, the selected test objects were a simple enough environment for us to infer and thoroughly characterize the interaction with the filter within the limits of our computational power while being representative of a real case scenario.

**FIGURE 4:**
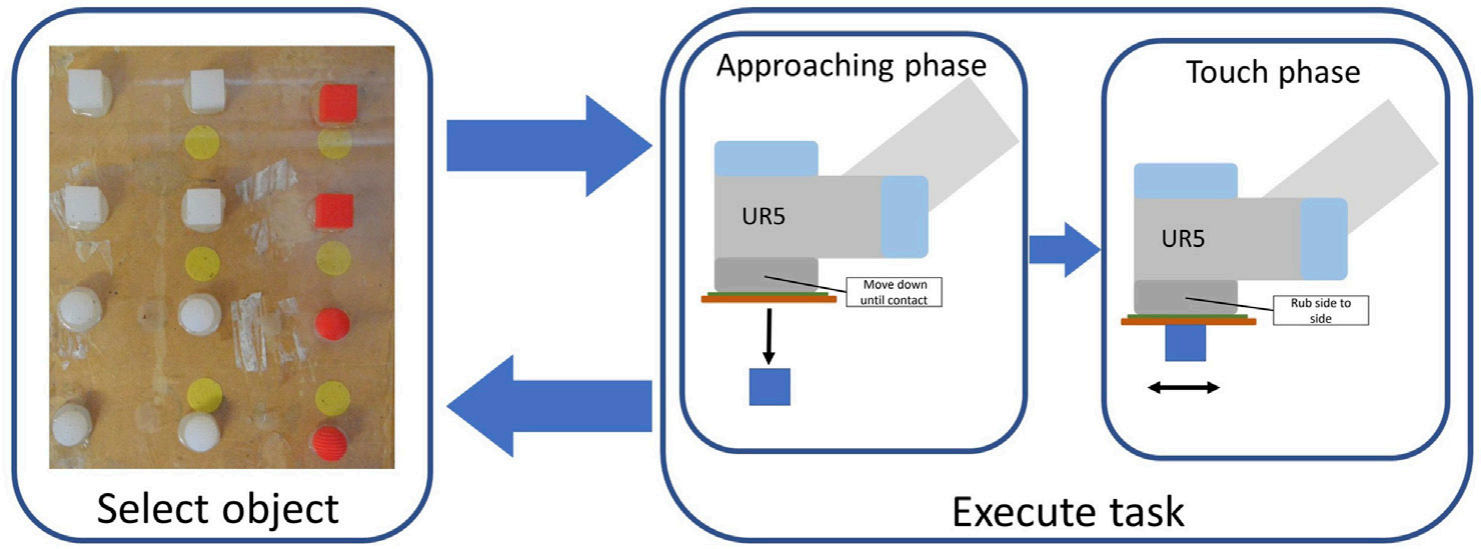
Work-flow of a single trial during data collection. First, the object of interest is selected among the twelve possible options: four combinations of shape and roughness for each of the three levels of stiffness. Next, the approaching and touch phases are performed on the object of interest.

Every object was subject to 50 trials for each of the four tested materials, for a total of 200 trials per object and 2,400 overall trials. Figure 4 illustrates the workflow implemented to collect data with our system once the filter was put in position. The same process was repeated for each tested material, leading to four different data-sets of the same 12 test objects that were analyzed and are compared in Section 3.

### 2.3 Data Processing

Before using the data for classification tasks, we decided to evaluate the quality of the tactile data by reducing their dimensionality and trying to observe spontaneous clustering behaviors. All the steps listed in this section were performed on the entire data-set and on the three subsets consisting of all the test objects of the same material.

First, we applied principal component analysis (PCA). This was used to strongly reduce the number of dimensions, from the initial 12,500 to a pre-selected set of retained principal components, where *p*_*i*_ is the *i*th principal component (Abdi and Williams, 2010). Then, we evaluated the information loss by analyzing what fraction of the original data variance was retained as a function of the number of considered principal components. Several other dimensionality reduction techniques could have been implemented for this purpose, such as t-SNE (Maaten and Hinton, 2008), but we decided to implement PCA because it is widely used in filter-based tactile sensing (Hughes et al., 2021).

Next, we analyzed the structure of the collected data, looking for class-dependent clusters. In the case of the full data-set, each one of the 12 objects corresponded to a different class, for a total of 12 classes. For the subsets, they only had four objects each, and thus, four different classes. Ideally, a good data structure has a low intra-class distance and a high inter-class distance. Hence, we used the silhouette score (Rousseeuw, 1987) to quantify the quality of the structure. The silhouette score is a good and compact approximation of the quality of the data structure, when dealing with a labeled data-set, that can provide a clear insight into how well classes are separated from each other. Moreover, such a metric has already been used in tactile information-based classification tasks (Scimeca et al., 2020a) and can be computed as follows:

$$s\left(i\right)=\frac{b\left(i\right)-a\left(i\right)}{max\left(a\left(i\right),b\left(i\right)\right)}$$ (1)

where *a*(*i*) and *b*(*i*) are the mean intra-class distance and the mean nearest-class distance for the object *i*, respectively. To effectively summarize the results, we computed the silhouette score *s* as the mean of the scores among all classes. Thus, the score *s* can assume values between -1 and 1 included, where higher values represent a better-defined data structure, which may potentially lead to a more accurate classification.

### 2.4 Classification

In order to understand how the difference in data structure affects the classification performance, we compared the performance of several machine learning algorithms (MLAs) while working on the four data-sets. Similar to Section 2.3, this process was performed both on the full data-set and on the three single-material subsets. We decided to implement nine standard MLAs: Nearest Neighbors (Goldberger et al., 2004), Linear SVM (Mayoraz and Alpaydin, 1999), RBF SVM (Yan et al., 2006), Gaussian process (KI Williams, 2006), Decision Tree (Steinberg and Colla, 2009), Random Forest (Cutler et al., 2012), Feed Forward Neural Network (Rumelhart et al., 1986), Naive Bayes (Manning et al., 2008), and QDA (Pedregosa et al., 2011). All the classifiers were implemented using the Scikit-learn library (version 0.32.1) in Python 3.8 (Pedregosa et al., 2011). In particular, the built-in functions were used with the default library parameters onto each data-set individually (Dragon Skin 30, Dragon Skin 20, Ecoflex 00–30, and Ecoflex 00–10). For the purpose of reproducibility, the following details about the MLAs are provided: the kernel used in the Gaussian process was “1.0*RBF(1.0)”, Decision Tree used the “gini” criterion, Random Forest used the ‘gini’ criterion and 10 estimators, Naive Bayes was implemented as a GaussianNB classifier, Nearest Neighbors used three nearest neighbors, Linear SVM and RBF SVM had a regularization parameter of 1, QDA used a tolerance of 10^–4^, and Neural Net was implemented as an MLP with one hidden layer of 100 neurons, activation “relu”, solver “adam”, and a maximum number of iteration of 1,000 or until convergence. The reason behind such a high number of different algorithms was that we did not want our conclusion to be biased by the classifier. Thus, by considering nine different ones, we were able to generalize our results and highlight the difference made by the structure and quality of the provided data rather than the intrinsic difference among the classifiers themselves. Parameters tuning has not been performed in order to maintain visible relative differences among the different data-sets and allow us to infer the effect of the data structure on the performance, rather than the MLAs’ architecture. Concerning the expected performance, we assume that higher quality in the tactile data would lead to a better classification in the majority of the cases. Because all classes are perfectly balanced with 50 samples per class, we use accuracy, as main metric to assess the performance of the classifier.

## 3 Results

This section showcases and analyzes the data structure and the classification performance of the four data-sets collected as described in Section 2.2. Firstly, we showcased the performance of commonly used silicones in tactile sensing and highlighted the differences that arise from the distinct mechanical properties of the tested materials. Secondly, we divided the test set based on the material of the test objects so as to showcase how the characteristic of the environment can affect the structure and performance obtained by the different filter’s materials. Finally, we show the structural damage experienced by the filters as a result of the mechanical interaction with the environment.

### 3.1 Filter-Mediated Response

**FIGURE 5:**
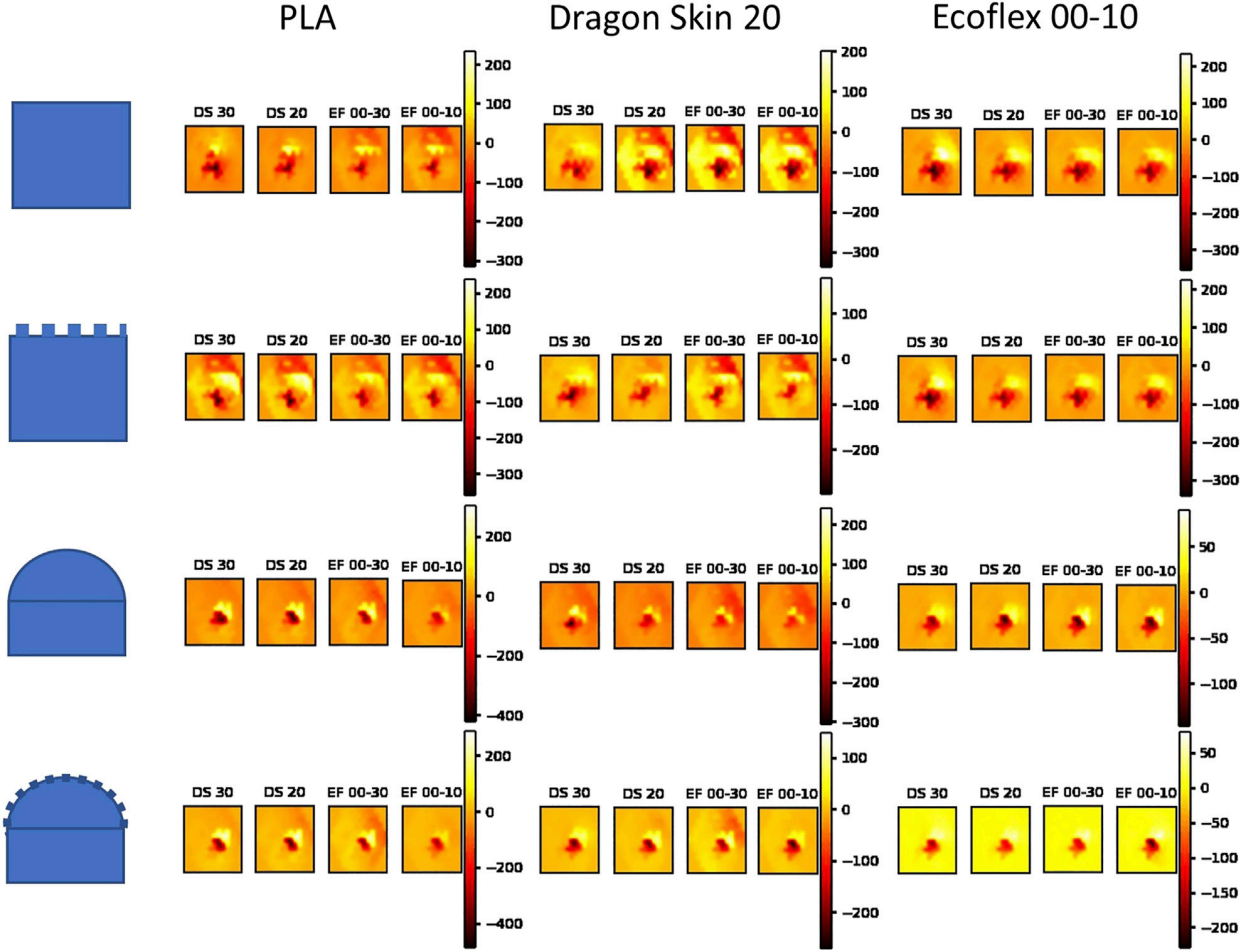
Raw tactile data as a function of filter’s material, and test object’s material, shape, and texture. Tactile images are taken after 2.5 s of the touch phase, performed as described in Section 2.2. For displaying purposes, Dragon Skin is abbreviated DS and Ecoflex as EF.

First, we show how the different filter’s materials affect the raw data picked up by the sensing unit: Figure 5 showcases the sensor recordings as a function of the filter’s material and the test object’s material, shape, and texture. Given the high number of dimensions, the raw data are not suitable for further analysis as they are, but they already show the general effect of filters with different values of shore hardness.

By analyzing images belonging to the same object, it is clear that the change in the filter’s material does not drastically change the overall response of the sensor, maintaining very similar spatial patterns and taxels’ ranges. Nevertheless, we easily noticed that more compliant materials, such as the Ecoflex series, promoted localized peaks of pressure, whereas stiffer materials tended to distribute the pressure over a larger area. This phenomenon is due to the mechanical properties of the materials that allow harder materials to propagate the load over a larger area, whereas softer materials tend to adapt to the shape of the object touched, showcasing more localized loads. Because the object is always touched using the same trajectory and all the filters have the same morphology, we assumed that the mechanical characteristics of the filter’s material were the determining factor in changing the morphological computation done by the filter itself, that, in turn, affected the processed signal recorded by the sensor.

### 3.2 Overall Results

**FIGURE 6:**
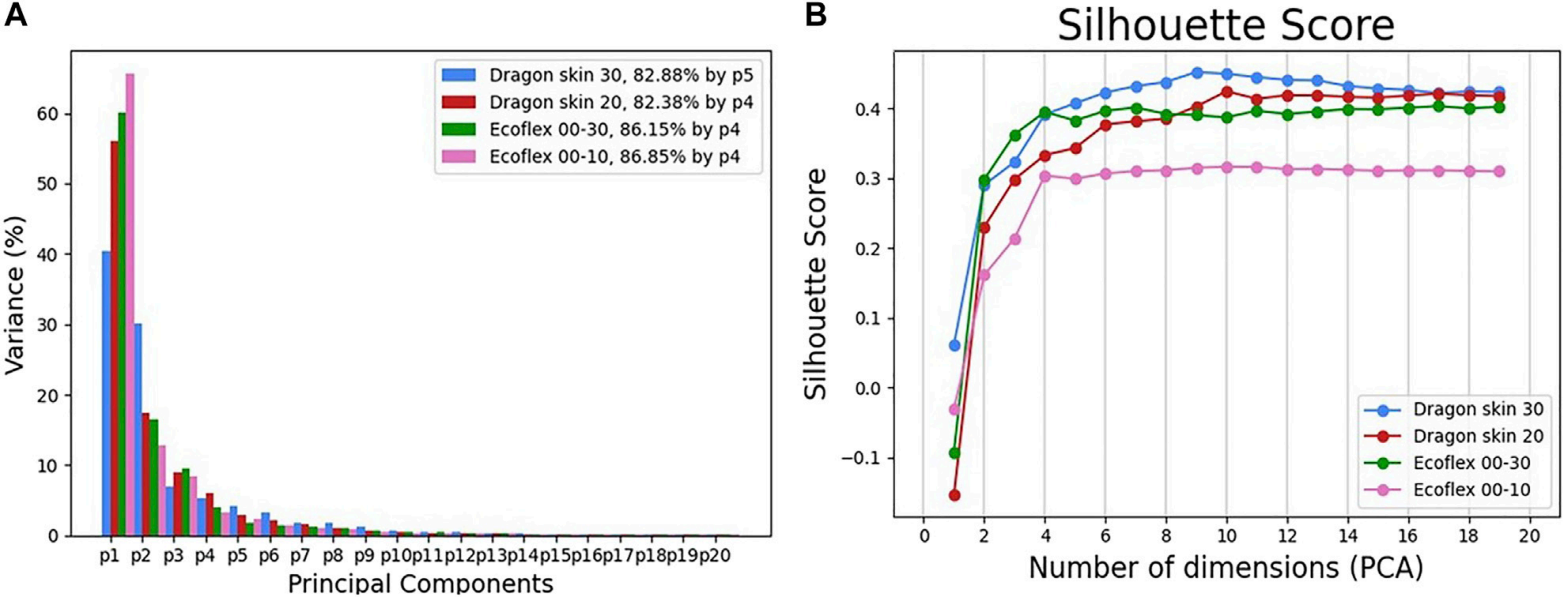
The variance retention of the principal components **(A)** and the silhouette score for increasing number of dimensions **(B)** for each filter’s material.

In order to define the starting point of our study and report the overall performance, we start by analyzing the full set containing all 12 test objects. The first step was reducing the number of dimensions of each data-point from the initial 12,500 to a small arbitrary pre-selected number thanks to a PCA. In order to check how much our choice of dimensionality reduction affects the tactile data, Figure 6A showcases the variance retention of the different materials as a function of the number of considered principal components.

The results showcase how the level of compliance of the filter’s material is proportional to the retained fraction of the initial data variance. This is especially noticeable in the first principal component *p*_1_ and the number of needed components to reach 80% of the initial variance: not only did Dragon Skin 30 need five principal components instead of four, but even the other three materials, whilst needing the same number of components, showed a compliance dependent cumulative value, with Ecoflex 00–10 reaching a high of 86.85%, followed by 86.15 % and 82.38% for Ecoflex 00–30 and Dragon skin 20, respectively.

Next, we studied the data structure. Because we considered the task of tactile classification, we opted to use the silhouette coefficient as a quality metric for the data structure itself, according to Section 2.3. Figure 6B illustrates the silhouette score as a function of the principal components and the filter’s material. Remarkably, we observed that the silhouette score was proportional to the stiffness of the material: more compliant materials consistently exhibited a worse silhouette score than stiffer ones, with Ecoflex 00–10 scoring significantly less than the other three materials, which appeared to be more clustered together. We can then assume that the ability to retain a higher fraction of the initial data variance does not imply a higher class separation, thus, a better structure of the data. The results clearly show that the best data structure is obtained when losing more of the initial variance: the embodied intelligence of the Dragon Skin 30 filter, combined with the PCA, can filter out unnecessary data variance, thus, highlighting signal features that can achieve a better class separation.

**FIGURE 7:**
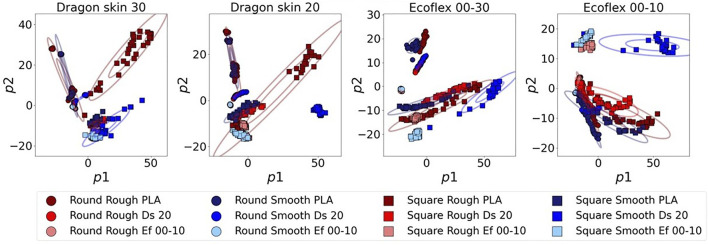
PCA projections of the first two principal components. The ellipses are drawn at one standard deviation and three standard deviations. “Ef” and “Ds” indicate Ecoflex and Dragon Skin, respectively.

In order to have a deeper insight into the class separation, we decided to plot the data-sets according to the first two principal components (see Figure 7).The plots clearly show how Ecoflex 00–10 is unable to obtain good separation of the classes, resulting in high overlapping of widely spread clusters. It is also noticeable that, even if the other three materials have similar silhouette scores, as previously discussed, the 2D PCA plots showcase very different behaviors: Ecoflex 00–30 has a homogeneous mild overlap of several classes, whereas the Dragon Skin series tends to have a good clustering of the majority of the classes, but completely fails to cluster few specific classes, such as “Square Rough PLA”.

**FIGURE 8:**
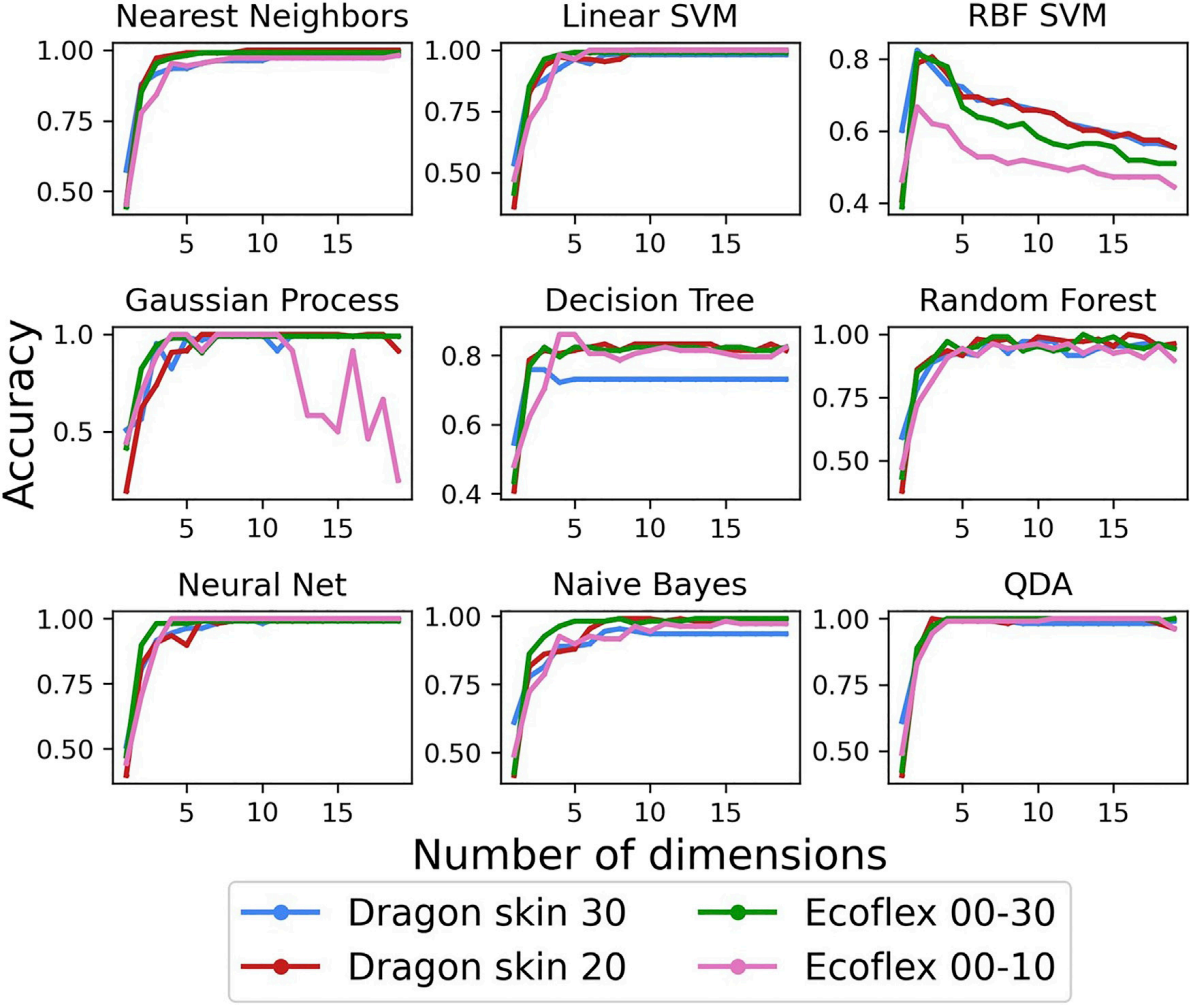
Classification accuracy using the nine different classifiers as a function of the PCA dimensions.

Finally, we focused on the tactile discrimination performance: we wanted to show how the differences in data structure affect the overall performance while performing tactile classification. As discussed in Section 2.4, we implemented nine different MLAs in order to avoid any classifier-specific bias and focus on the effect of the data on the classification performance. Figure 8 shows the performance of the MLAs as a function of the principal components for each of the tested filter’s material. Accuracy was selected as a valid metric for the performance because of the perfect balance of the classes, having 50 data-points for each class in each data-set. Specifically, we were interested in two features of the resulting accuracy: the value of the reached plateau as the number of retained principal components increases and the transient that leads up to such plateau.

The first obvious result was that Ecoflex 00–10 performed, on average, worse than the other materials, having both a lower plateau and a slower transient. We assumed that the worse data structure observed in Figure 6B and Figure 7 is the reason behind the worse performance in terms of classification accuracy. The other three materials, which also showcased a very similar silhouette score, had very similar performance, with some exceptions: Dragon Skin 30 showcased a poor performance both in Decision Tree and Naive Bayes, whereas Ecoflex 00–30 fell short in RBF SVM. All in all, Dragon Skin 20 seemed to have the best performance, only showcasing a slower transient in three out of nine classifiers but always reaching the highest plateau by the 10th principal component. In summary, Dragon Skin 20 was the only filter that maintained the highest accuracy in all nine MLAs, whereas the others performed significantly worse in at least one of them. In conclusion, despite its higher silhouette score, the stiffer Dragon Skin 30 showcased a worse classification accuracy, leading us to believe that the optimal stiffness for our tactile filter is not as high as possible, but it lays somewhere in the middle of the Dragon Skin series.

### 3.3 Material Based Results

Starting from the overview provided in Section 3.2, we divided the data-sets into three different subsets depending on the material of the test objects, and we analyzed the environment’s effect on the filters’ relative performance. The aim of this section is to understand how the mechanical properties of the surrounding environment can affect the performance of each individual filter, altering the relative performance among them. Understanding the relationship between the filter’s and the environment’s properties can lead to more insight regarding the choice of filter’s material in order to achieve the best data structure and classification accuracy as a function of the environment.

**FIGURE 9:**
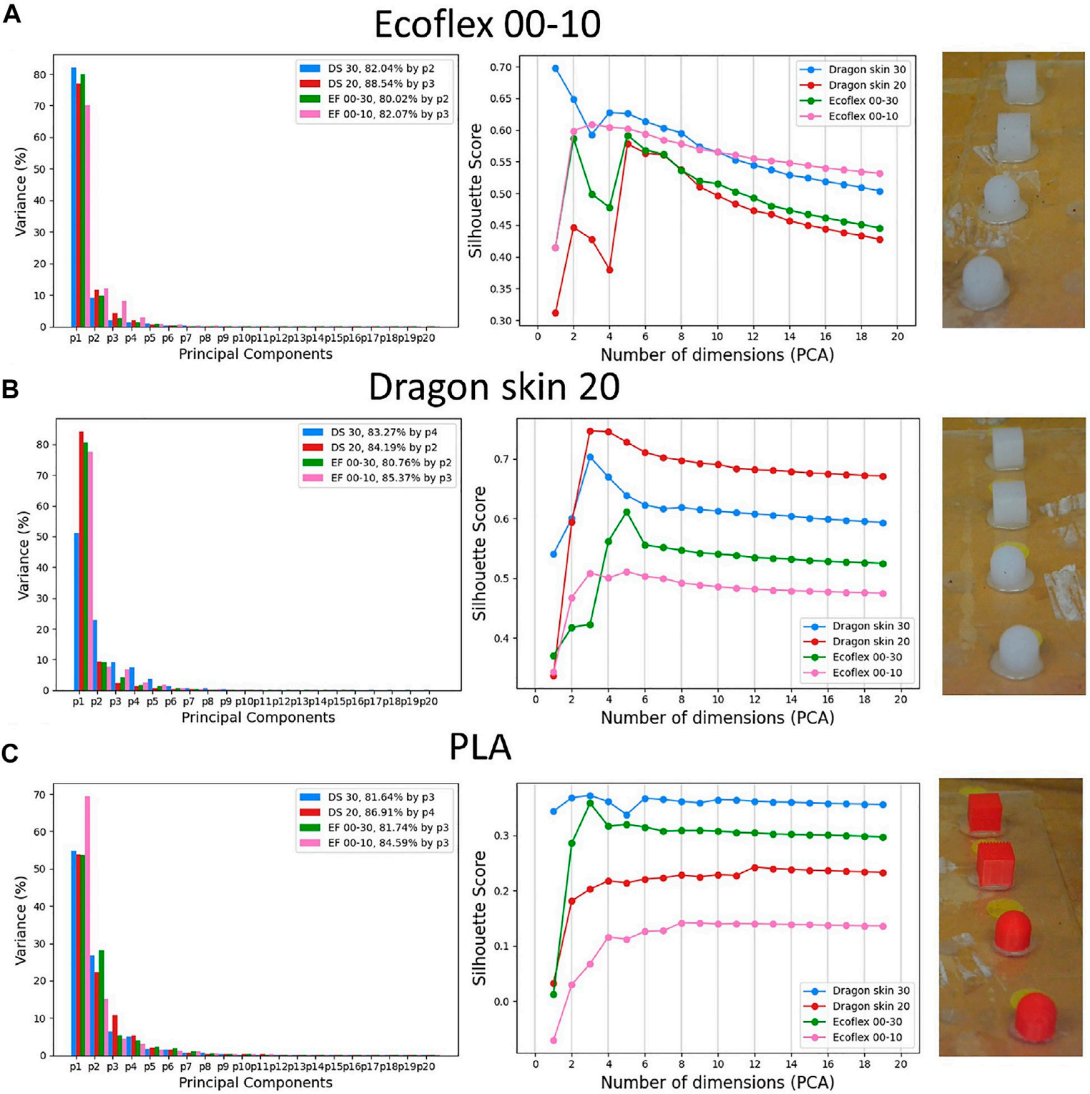
PCA variance retention and silhouette score when testing the filter’s material on the three different subsets made by test objects of the same material: Ecoflex 00–10 **(A)**, Dragon Skin 20 **(B)**, and PLA **(C)**.

First, we investigated how the variance retention and the silhouette score would change when considering the three different subsets individually. Figure 9 summarizes such results.

First, we noticed that, unlike Figure 6B, the silhouette scores of both the Ecoflex 00–10 and the Dragon Skin 20 filters did not asymptotically tend to their maximum, but rather decreased as the number of retained principal components increased. We believed that this was due to the amount of noise present in each principal component: having fewer classes in the test set and having good mechanical coupling between the filter and environment, since they are both manufactured using silicone, lead to a good separation of the classes with few components. Then, if we kept adding principal components, we eventually started adding the data variance that was produced by noise, thus, the silhouette scores started decreasing. Concerning the Ecoflex 00–10 subset (see Figure 9A), it was clear that the data structure indicated Ecoflex 00–10 as the best filter material, given its higher silhouette score starting from 10 principal components onward. The reason behind this could be that the coupling between filter and environment of the same material produces less overall noise: less noise in the recorded signal eventually results in a slower decay of the silhouette score as the number of principal components, and therefore signal variance, increases. Especially when compared with the full set results in Figure 6, we noticed a strong increase in the quality of the Ecoflex 00–10 data structure, making such material move from worst to best in terms of silhouette score. Similarly, when considering the Dragon Skin 20 subset, it was clear that the Dragon Skin 20 filter was able to obtain a better data structure, consistently having a higher silhouette score than the other ones. Concerning the PLA subset, we did not have a PLA filter, but we could see how the stiffest material among the ones tested, Dragon Skin 30, was able to produce a better data structure, denoted by a higher silhouette score. Nevertheless, the compliance of Dragon Skin 30, and, therefore, the compliance of all the tested silicones, was much higher than PLA’s, resulting in much lower overall values of the silhouette score. Regarding the PCA variance retention, we could see how it was not proportional to the material’s compliance in the case of smaller subsets consisting of objects made of one material only. Ecoflex 00–10 showed low retention, relatively to the other filter’s materials when tested on Ecoflex 00–10 objects, and a relatively high one when tested on the PLA subset. On the other hand, Dragon Skin 20 showcased its highest variance retention when tested on the object of the same material. Predictably, the tested materials have low retention when touching a material such as PLA, which is much stiffer than silicones. Overall, the results indicated that in order to achieve a good data structure, the tactile filter should match the mechanical characteristics of the environment as much as possible.

**FIGURE 10:**
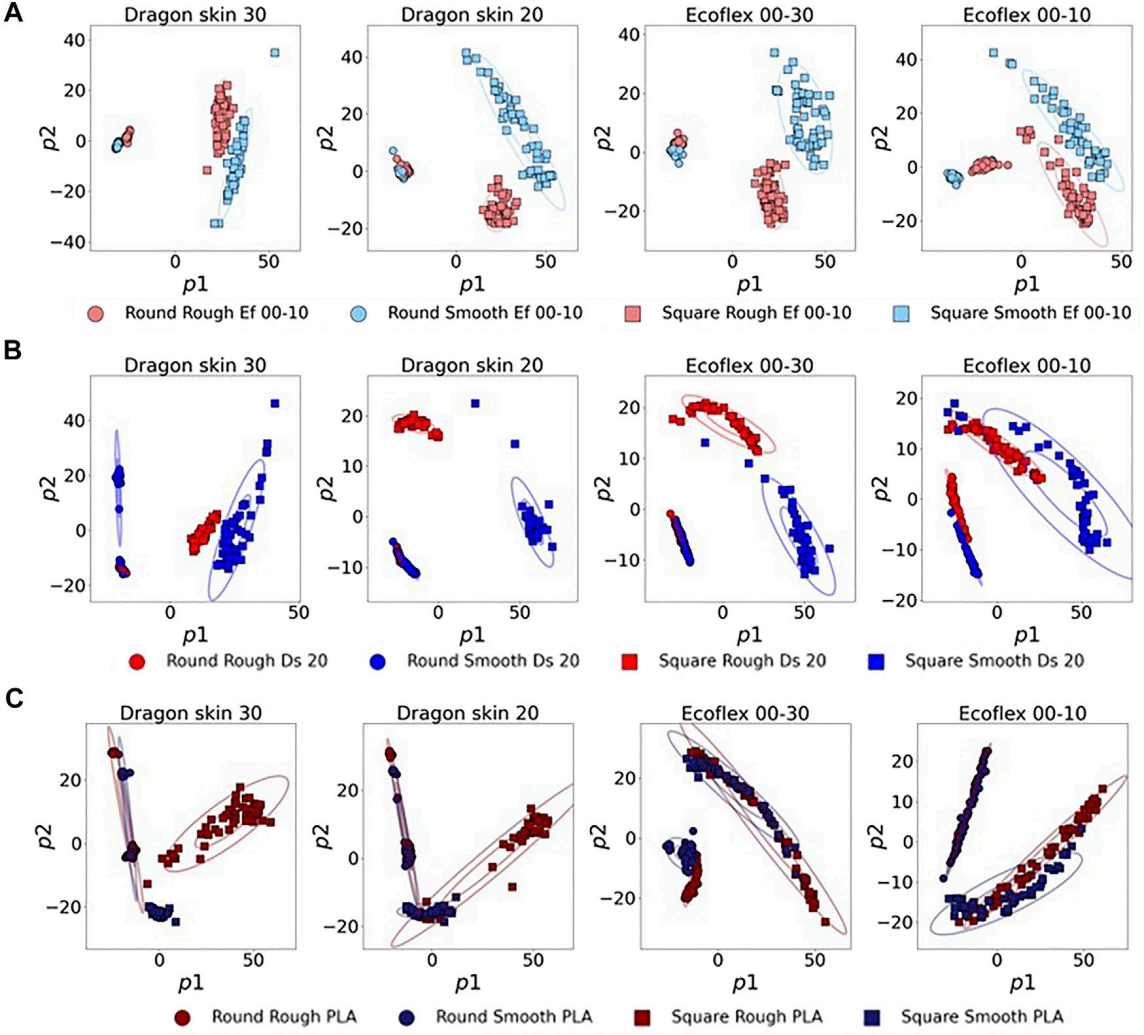
2D variance retention plots when testing the filter’s material on the three different subsets made with test objects of the same material: Ecoflex 00–10 **(A)**, Dragon Skin 20 **(B)**, and PLA **(C)**. ‘Ef’ and ‘Ds’ indicate Ecoflex and Dragon Skin, respectively.

In order to have a better insight into what the data structure obtained by the different filter’s materials looks like, we plotted the 2D scatter plots, using the first two principal components of the PCA. Figure 10 illustrates such plots for the three tested subsets. Starting from the Ecoflex 00–10 subset, we could clearly see that only the Ecoflex 00–10 filter was able to fully separate ‘Round Rough Ef 00-10’ and ‘Round Smooth Ef 00–10’, which were clustered together while using any other filter’s material. In the case of the Dragon Skin 20 subset, we noticed how the filter made with the same material, Dragon Skin 20, was able to obtain more compact clusters, especially considering the two square classes. However, we could see that the two round classes completely overlapped when considering only the first two principal components: this overlapping could be probably resolved by using the third principal component, as can be deduced by the increasing slope of the Dragon Skin 20 silhouette score in Figure 9B. As predicted, the PLA subset showcased greater levels of class overlapping and class spread. Nevertheless, both overlapping and spread were strongly reduced while using the Dragon Skin 30 filter, leading to a higher silhouette score (see Figure 9C).

**FIGURE 11:**
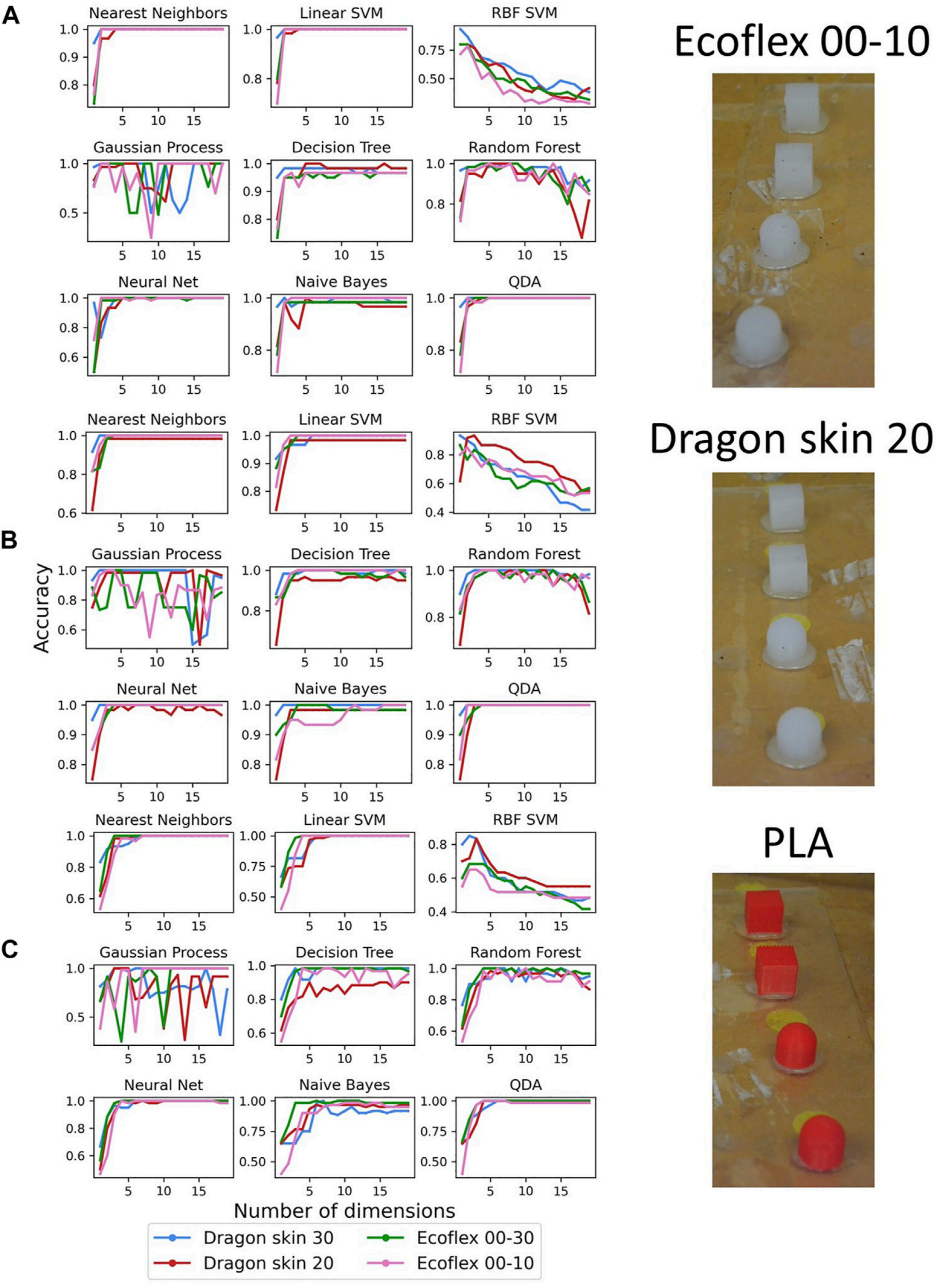
Classification accuracy results when testing the filter’s material on the three different subsets made by test objects of the same material: Ecoflex 00–10 **(A)**, Dragon Skin 20 **(B)**, and PLA **(C)**.

Last, we analyzed how the different data structures affected the classification accuracy for each individual subset (see Figure 11).

When using the Ecoflex 00–10 subset, the Ecoflex 00–10 filter showcased very sharp transients and overall high accuracy in almost all classifiers, leaving out only RBF SVM. When compared with its performance in Figure 8, we observed much steeper transients, indicating a good performance even with a small number of retained principal components. Similarly, the Dragon Skin 20 filter showed highly reduced transients and good accuracy when tested on the Dragon Skin 20 test set for all classifiers except Decision Tree. In the case of RBF SVM, we could also see that it outperformed every single one of the other materials. However, because of its high accuracy in the overall data-set, it was not possible to detect any obvious increase in accuracy. Concerning the PLA subset, we knew that the quality of the data structure was low for all filter’s materials (see Figure 9C), and we could not observe any noticeable difference in classification performance among the data-sets belonging to different filter’s materials. Even if Dragon Skin 30 was shown to have the better data structure, it did not end up playing an important role when performing tactile classification, except for the case of Naive Bayes, in which we appreciated a very sharp transient, especially if compared with the other materials. Noticeably, Gaussian process seemed to have inconsistent accuracy in all cases, especially when compared with Figure 8, and we believed that this was due to the smaller size of the data-sets, negatively affecting the choice of the kernel. Overall, the results suggested once again that matching the environment’s mechanical characteristics produces optimal results, even if slightly, whereas filters of significantly higher or lower compliance inevitably lead to worse performances. We believe that these experimental findings were caused by particular conditions of the trade-off between compliant contact and mechanical filtering of the signal. As previously reported in the literature (Shimojo, 1997), a compliant filter is able to better adapt to the environment, achieving a larger contact area, but also acts as a low-pass mechanical filter, losing the high-frequency components of the acquired data. However, when the filter has the same compliance as the environment or less, it does not affect the recorded signal because its cut-off frequency is higher than what the dynamics of the environment can produce. Therefore, matching the visco-elastic properties of the environment is the best design choice for tactile filters because it maximizes the compliance of the filter, thus increasing the contact area with the environment, without introducing information losses.

### 3.4 Damage

**FIGURE 12:**
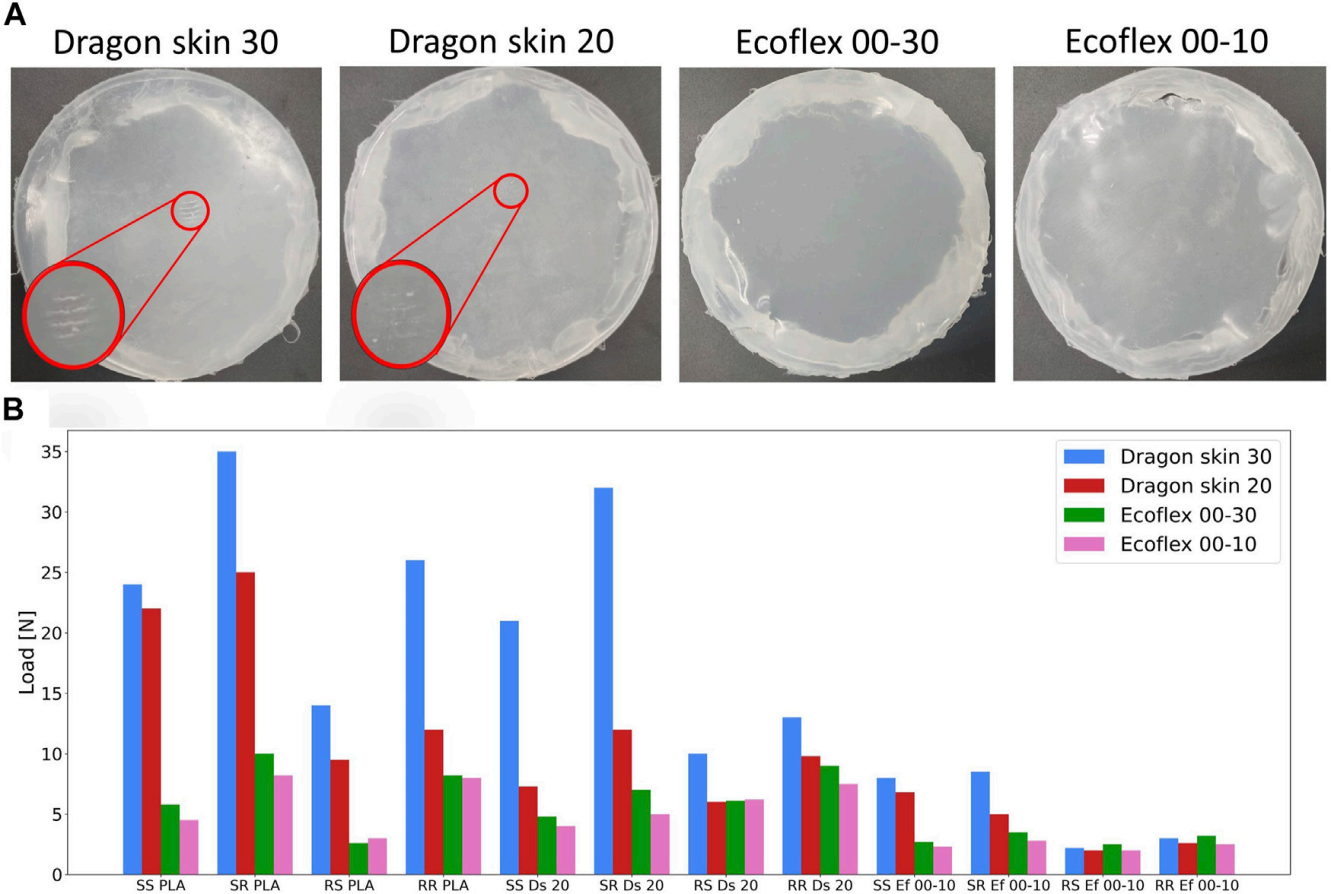
Tactile filters after the end of the experimental trials **(A)**. Any damage on the outer diameter is due to the removal of the filter from the end-effector and not from the contact with the test set. Measured load applied on each filter while performing the experiment **(B)**. Objects’ names are as follows: “SS” is Square Smooth, “SR” is Square Rough, “RS” is Round Smooth, “RR” is Round Rough, “Ds” is Dragon Skin, and “Ef” is Ecoflex.

Finally, we evaluated by visual inspection the condition of the filters after the experiments. Figure 12A shows the state of all four filters after the end of the 2,400 experimental trials. The outcome clearly shows that stiffer filters are more prone to mechanical damage, especially when coming into contact with even stiffer objects like the PLA subset. In the Dragon Skin 30, a plastic deformation caused by the groove on top of both the rough PLA objects was clearly visible. The same pattern was also observable in the Dragon Skin 20 filter, even if much less visible, whereas the Ecoflex series did not show any sign of plastic deformation or mechanical damage under visual inspection. Any damage to the outside diameter of the filter was due to the removal of the filter itself from the robot and not from the interaction with the environment. Probably, the damage was caused by the higher load exerted on the filter while operating with harder materials: the approaching phase is stopped when a given load was detected by the sensing unit (see Section 2.2). As already discussed, harder materials could lead to a bigger load on the filter itself to achieve the same tactile signal. To verify such a hypothesis, we ran the experimental protocol a second time, performing only one trial per object, while recording the exerted load at the end of the approaching phase using a scale (*CBC bench counting scale, Adam United Kingdom LTD.*) placed below the testing set. Figure 12B shows that when using filters made of Dragon Skin, especially Dragon Skin 30, the contact was detected only at a greater load, confirming that harder materials indeed behaved as mechanical filters. In turn, a greater load led to greater damage. The damage of the filter might not matter for short experiments, but it is an important drawback for longer ones, leading to the conclusion that softer materials should be preferable for long-term solutions, provided that they provide similar or just slightly worse classification performances. As a result, when sensing stiff objects, we came across a trade-off between the data quality and the life span of the system: matching the high stiffness would increase the quality of the data structure and the classification accuracy, but it would also shorten the life span of the tactile filter, promoting damage and plastic deformation.

## 4 Discussion

In this work, we tried to characterize the relationship between the environment and filter-based tactile sensing. We believe that the surrounding environment plays an important role in dictating the requirements for tactile filters. We not only aimed to investigate the relationship between those materials and the environment but also wanted to allow soft robots to fully utilize the potential offered by tactile sensing; therefore, we decided to use as tactile filters the four silicones commonly used in soft robotics: Dragon Skin 30, Dragon Skin 20, Ecoflex 00–30, and Ecoflex 00–10.

To create a starting point for our analysis and test the performance of the different filter’s materials, we acquired data from a set of 12 objects, showcasing different shapes, roughness, and materials. For each object and each filter’s material, we performed 50 trials, for a total of 2,400, using a UR5 robotic arm to carry the load of the sensor unit and the tactile filter and to perform active touch on the test objects. By analyzing the PCA variance retention and the silhouette score, we discovered that variance retention is proportional to the filter’s compliance, and a more separable data structure is proportional to the filter’s stiffness instead. Moreover, we showed how the best classification accuracy was achieved by Dragon Skin 20, outperforming the other materials in eight machine learning classifiers out of nine.

Then, in order to break down the relationship between the mechanical properties of the filter and the ones of the environment, we divided the test set into three subsets based on the material of the test object: one subset containing only Ecoflex 00–10 object, one only Dragon Skin 20, and the last one PLA. After performing the same data analysis on all subsets, we discovered that matching the mechanical characteristics of the environment leads to a better data structure and, in turn, to a higher classification accuracy: both the Ecoflex 00–10 and the Dragon Skin 20 filters outperformed the other filter’s materials when tested with the single-material subset of the same material. When tested using the PLA object, all materials scored a very low silhouette score, but Dragon Skin 30 had the highest value: we believe that this was due to Dragon Skin 30 being the stiffest material out of the four tested materials; therefore, the closest to the much stiffer PLA. However, no noticeable difference was observed between different materials in classification accuracy. We believe the observed behavior was due to a combination of limited losses on the high frequency components of the signal and a compliant as possible contacting surface. Finally, we showed how stiffer materials are more prone to damage, making them unsuitable for long-term applications.

Overall, we utilized four common silicones as tactile filters to investigate the relationship between the filter’s and the environment’s mechanical characteristics, paving the way for environment-specific filter-based tactile sensing in soft robotics. We proved that matching the compliance of the surrounding environment is the best strategy to achieve better data structure and higher accuracy. However, we acknowledge that our work is limited to a finite set of filter’s and environment’s materials. We believe that these findings can be extended to the other products of the Ecoflex and Dragon Skin series and possibly to silicones in general, effectively covering most soft robotics applications, but cannot be generalized for all materials. Because of such limitations, we endorse future work on a wider range of materials and the possible development of a mathematical model to analytically represent the relationship between tactile filters and surrounding environments in terms of data structure and classification accuracy.

## Data Availability

The original contributions presented in the study are included in the article/Supplementary Material; further inquiries can be directed to the corresponding author.
